# Inhibition of elongin C promotes longevity and protein homeostasis via HIF‐1 in *C. elegans*

**DOI:** 10.1111/acel.12390

**Published:** 2015-09-11

**Authors:** Wooseon Hwang, Murat Artan, Mihwa Seo, Dongyeop Lee, Hong Gil Nam, Seung‐Jae V. Lee

**Affiliations:** ^1^ Department of Life SciencesPohang University of Science and TechnologyPohangGyeongbuk37673South Korea; ^2^ Information Technology Convergence EngineeringPohang University of Science and TechnologyPohangGyeongbuk37673South Korea; ^3^ School of Interdisciplinary Bioscience and BioengineeringPohang University of Science and TechnologyPohangGyeongbuk37673South Korea; ^4^ Center for Plant Aging ResearchInstitute for Basic ScienceDaegu42988South Korea; ^5^ Department of New BiologyDGISTDaegu42988South Korea

**Keywords:** aging, *C. elegans*, elc‐1, hypoxia‐inducible factor 1, protein homeostasis

## Abstract

The transcription factor hypoxia‐inducible factor 1 (HIF‐1) is crucial for responses to low oxygen and promotes longevity in *Caenorhabditis elegans*. We previously performed a genomewide RNA interference screen and identified many genes that act as potential negative regulators of HIF‐1. Here, we functionally characterized these genes and found several novel genes that affected lifespan. The worm ortholog of *elongin C*,* elc‐1*, encodes a subunit of E3 ligase and transcription elongation factor. We found that knockdown of *elc‐1* prolonged lifespan and delayed paralysis caused by impaired protein homeostasis. We further showed that *elc‐1* RNA interference increased lifespan and protein homeostasis by upregulating HIF‐1. The roles of elongin C and HIF‐1 are well conserved in eukaryotes. Thus, our study may provide insights into the aging regulatory pathway consisting of elongin C and HIF‐1 in complex metazoans.

## Introduction

Proper levels of oxygen are essential for the survival of aerobic organisms. Hypoxia‐inducible factor 1 (HIF‐1) is a key transcription factor that governs cellular responses to low oxygen (reviewed in Semenza, 2012). In normal oxygen conditions, HIF‐1 is hydroxylated by the proline hydroxylase EGL‐9. This leads to the ubiquitination and degradation of HIF‐1 by an E3 ligase containing the von Hippel–Lindau‐1 (VHL‐1) tumor suppressor. VHL‐1 determines the substrate specificity of the E3 ligase. In contrast, hypoxia or inhibition of EGL‐9 or VHL‐1 promotes the stabilization of HIF‐1. Stabilized HIF‐1 translocates to the nucleus and regulates the transcription of genes that control hypoxic responses. The crucial functions of human HIF‐1 are highlighted by the findings that HIF‐1 is associated with various diseases and pathological conditions, including cancer, arterial diseases, and organ transplant rejection (reviewed in Semenza, 2012).

*C. elegans* HIF‐1 also plays key roles in various physiologic processes, including stress and pathogen responses, axon guidance, iron and protein homeostasis, and reproduction (Pocock & Hobert, 2008; Bellier *et al*., 2009; Mehta *et al*., 2009; Zhang *et al*., 2009; Powell‐Coffman, 2010; Romney *et al*., 2011; Ackerman & Gems, 2012; Fawcett *et al*., 2015). Recent studies demonstrate that HIF‐1 activation promotes longevity in *C. elegans* (Mehta *et al*., 2009; Muller *et al*., 2009; Zhang *et al*., 2009; Lee *et al*., 2010; Leiser *et al*., 2011; Hwang *et al*., 2014). However, the components that mediate longevity in response to HIF‐1 activation remain unclear.

In our previous report, we performed a genomewide RNA interference (RNAi) screen using an HIF‐1 reporter, *nhr‐57p::gfp* transgenic *C. elegans*. We found 245 putative HIF‐1 regulators (Lee *et al*., 2010; Fig. S1, Supporting information). Here, we characterized the functions of these potential HIF‐1 regulatory genes in lifespan regulation. We found six genes whose knockdown increased the lifespan of worms. Among those, knockdown of *elc‐1*, which encodes a worm homolog of elongin C, lengthened lifespan by stabilizing HIF‐1. In addition, genetic inhibition of ELC‐1 increased protein homeostasis in a HIF‐1‐dependent manner. Elongin C is evolutionarily conserved and therefore may affect aging in complex animals, such as mammals as well as *C. elegans*.

## Results

**Figure 1:**
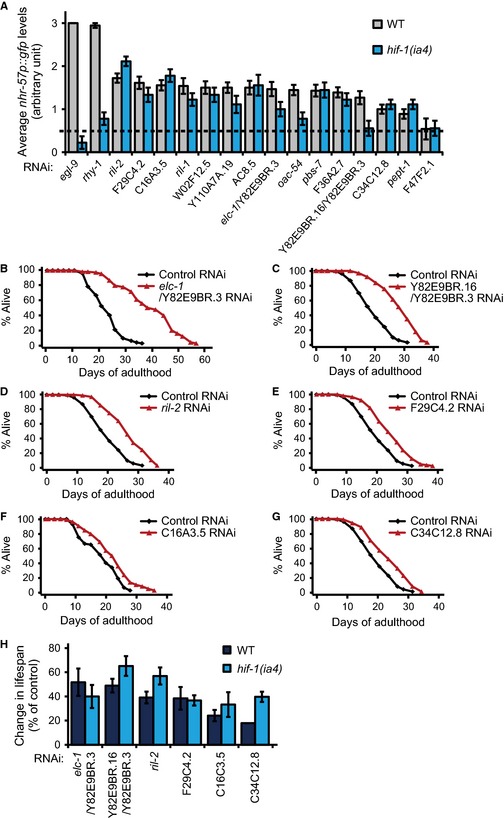
Semiquantification of *nhr‐57p::gfp* levels and the effects of *nhr‐57* inducer RNAi clones on lifespan. (A) Among 53 candidate RNAi clones selected from our previous screen in a liquid culture system (Lee *et al*., 2010), 16 RNAi clones consistently increased the level of *nhr‐57p::gfp* in a solid culture system, mostly in a *hif‐1‐*independent manner. The arbitrary cutoff value was 0.5 as indicated by a dotted line. *egl‐9* RNAi was used as a positive control. Error bars indicate standard error of the mean (SEM) (*n* > 12). (B–G) Lifespan curves of wild‐type (WT) animals treated with commercially available RNAi clones targeting *elc‐1*/Y82E9BR.3 (B), Y82E9BR.16/Y82E9BR.3 (C), *ril‐2* (D), F29C4.2 (E), C16A3.5 (F), or C34C12.8 (G). Lifespan assays were performed at least twice independently. See Fig. S3 for the results of lifespan assays upon treating with other *nhr‐57p::gfp* inducer RNAi clones that did not increase lifespan. (H) Percent changes in the lifespan of WT and *hif‐1* mutant worms after treatment with RNAi clones shown in Figs 1B–G and S4. The mean lifespan was compared with those of control RNAi‐treated worms in at least two trials, and error bars indicate SEM. See Table S2 for additional trials and statistical analysis for lifespan data shown in this figure.

Our previous genomewide screen was performed in a liquid culture system (Lee *et al*., 2010); however, conventional lifespan assays are performed in solid culture systems. Therefore, we re‐examined 53 RNAi clones that were strong *nhr‐57p::gfp* inducers and found 16 RNAi clones that robustly increased *nhr‐57p::gfp* levels in the solid culture system [Fig. S1, Table S1, Supporting information, and Fig. 1A. Note: Commercially available RNAi clones that were designed to target *elc‐1* and Y82E9BR.16 have another common target, Y82E9BR.3. We therefore designated these two RNAi clones as *elc‐1*/Y82E9BR.3 RNAi and Y82E9BR.16/Y82E9BR.3 RNAi (Fig. S2, Supporting information)]. Surprisingly, we found that 12 of the 16 RNAi clones induced *nhr‐57* independently of HIF‐1 (Fig. 1A). These data suggest that factors other than HIF‐1 also regulate the induction of *nhr‐57*.

Next, we performed lifespan assays with the 16 strong *nhr‐57* inducer RNAi clones and found that six RNAi clones significantly increased lifespan (Fig. 1B–H, Fig. S3, and Table S2, Supporting information). RNAi targeting *elc‐1,* a worm homolog of mammalian elongin C, and Y82E9BR.3, a worm homolog of ATP synthase subunit C, significantly promoted longevity (Fig. 1B,H). Likewise, RNAi targeting Y82E9BR.16, a worm homolog of solute carrier family 22 member 21, and Y82E9BR.3 promoted longevity (Fig. 1C,H). In addition, knockdown of the nematode‐specific gene *ril‐2* increased lifespan (Fig. 1D,H); this result is consistent with those presented in a previous report (Hansen *et al*., 2005). We also found that knockdown of the mitochondrial genes F29C4.2 (a worm homolog of cytochrome C oxidase subunit 6C), C16A3.5 (a worm homolog of NADH dehydrogenase [ubiquinone] 1 β subcomplex subunit 9), and C34C12.8 (a worm homolog of mitochondrial GrpE) extended lifespan (Fig. 1E–H). These results are consistent with many reports showing that mild inhibition of mitochondrial components confers longevity (reviewed in Van Raamsdonk & Hekimi, 2010; Hwang *et al*., 2012). We then examined whether the longevity caused by these six RNAi clones was dependent on HIF‐1. The RNAi clone that targeted *elc‐1*/Y82E9BR.3 was the only one that increased lifespan in a slightly *hif‐1*‐dependent manner (Figs 1H and S4, Supporting information). Together, these data indicate that RNAi clones that increase lifespan and induce *nhr‐57* expression levels do not necessarily act through HIF‐1.

**Figure 2:**
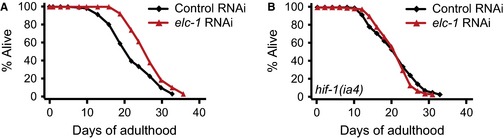
The effect of RNAi targeting *elc‐1* on lifespan. (A and B) Lifespan curves of wild‐type (A) and *hif‐1(ia4)* mutants (B) that were treated with an in‐house RNAi clone specifically targeting *elc‐1*. See Table 1 and Table S2 for additional information for lifespan data shown in this figure. The lifespan‐extending effect of *elc‐1* RNAi was modest (+12% on average) compared to that of *vhl‐1* mutation (+12% to +62%, Mehta *et al*., 2009). However, the lifespan‐extending effect of *elc‐1* RNAi is actually comparable to that of *vhl‐1* RNAi on wild‐type (+11%, Mehta *et al*., 2009). Please note that we were unable to determine the lifespan of *elc‐1* deletion mutants because they display a lethal phenotype.

Because RNAi clones against *elc‐1*/Y82E9BR.3 and Y82E9BR.16/Y82E9BR.3 had a common target gene, we generated RNAi clones targeting individual genes (see Figs S2 and S5, Supporting information for detailed description). We found that *elc‐1‐*specific knockdown increased lifespan in a largely *hif‐1*‐dependent manner (in five of nine trials) (Fig. 2A,B Table 1 and Table S2). Based on these results, we focused on the regulation of HIF‐1 by ELC‐1.

**Table 1:** Summary of lifespan results

Strain	Mean lifespan ± SEM (days)	% change	Total number of worms
WT control	20.7 ± 0.5		1960
*elc‐1(RNAi)*	23.1 ± 0.6	+12	1186
*elc‐1/*Y82E9BR.3*(RNAi)*	33.6 ± 2.8	+63	425
Y82E9BR.16*(RNAi)*	21.3 ± 2.5	+3	275
Y82E9BR.16/Y82E9BR.3*(RNAi)*	33.4 ± 0.8	+62	250
Y82E9BR.3*(RNAi)*	28.0 ± 0.2	+35	192
*hif‐1(ia4)* control	20.3 ± 0.6	−2	1751
*elc‐1(RNAi); hif‐1(ia4)*	21.1 ± 0.7	+4	1181
*elc‐1/*Y82E9BR.3*(RNAi); hif‐1(ia4)*	31.7 ± 1.4	+56	425
Y82E9BR.3*(RNAi); hif‐1(ia4)*	27.5 ± 0.4	+35	176

This table contains summary of the lifespan results in this study except lifespan screen data. Percent changes in mean lifespan of RNAi‐treated wild‐type and mutant animals were calculated against control RNAi‐treated wild‐type and mutant worms, respectively. See Table S2 for the results of each trial and statistical analysis for lifespan data.

**Figure 3:**
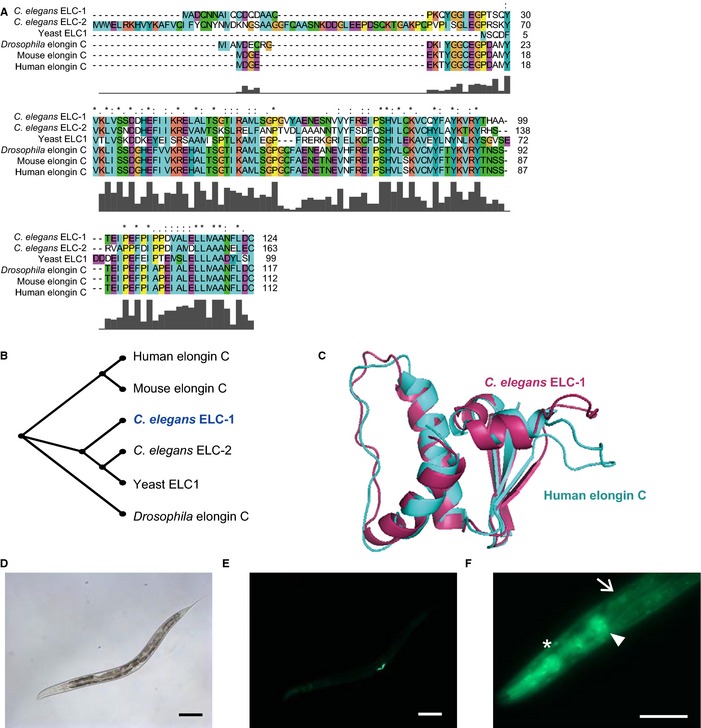
Evolutionary conservation and expression pattern of ELC‐1. (A) Alignment of amino acid sequences of *C. elegans* ELC‐1, *C. elegans* ELC‐2, yeast ELC1 (38% identities), *Drosophila* elongin C (79% identities), mouse elongin C (75% identities), and human elongin C (75% identities). The sequence identity values were calculated by comparing each sequence to that of *C. elegans* ELC‐1. BLAST was conducted using Clustal W and revisualized using Clustal X2 (Larkin *et al*., 2007). ‘*’ indicates an identical residue. ‘:’ indicates a residue that has conserved amino acids with strong similarities. ‘.’ indicates a residue that has conserved amino acids with weak similarities. Gray bars indicate scores for evolutionary conservation (Thompson *et al*., 1997). (B) A phylogenetic tree of elongin C in various species. This tree was generated using Clustal W2 and revisualized by Phylowidget (Larkin *et al*., 2007; Jordan & Piel, 2008). (C) Alignment of predicted human elongin C and *C. elegans* ELC‐1 protein structures displays strong similarities. (D) Bright‐field image of an *elc‐1p::elc‐1::gfp* animal shown in panel E. (E, F) *elc‐1p::elc‐1::gfp* was strongly expressed in the vulval muscle (E) and detected in the intestine (arrow), pharynx (arrowhead), and hypodermis (asterisk) after a longer exposure (F). Scale bar = 100 μm. Young adult worms were used for these images.

ELC‐1 is a worm homolog of mammalian elongin C. The amino acid sequences and structures of these proteins are well conserved from yeast to humans (Fig. 3A–C). Elongin C has two distinct roles. First, elongin C acts as a component of a transcription elongation factor in association with elongin A and elongin B (Bradsher *et al*., 1993a,b; Shilatifard *et al*., 2003). Second, elongin C functions as a component of an E3 ubiquitin ligase by binding to other components, including elongin B and pVHL; this complex determines the specificity for HIF‐1α degradation (Duan *et al*., 1995; Kim & Kaelin, 2003). Although mammalian elongin C has been functionally characterized, it is unknown whether *C. elegans elc‐1* regulates HIF‐1 and modulates HIF‐1‐dependent phenotypes.

We generated GFP‐fused *elc‐1‐*expressing transgenic animals to determine the expression patterns of *elc‐1*. We detected bright expression of ELC‐1::GFP in the vulval muscle and dim expression in the pharynx, hypodermis, and intestine (Fig. 3D–F). We found that ELC‐1 localized to both the cytoplasm and the nucleus (Fig. 3F). This result is consistent with the dual roles of mammalian elongin C as a transcription elongation factor and component of an E3 ligase.

**Figure 4:**
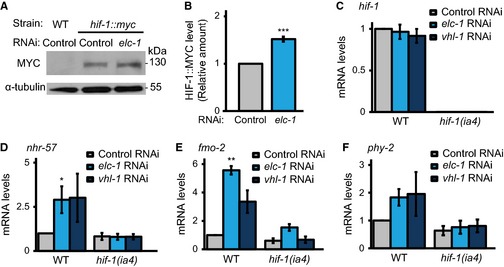
ELC‐1 modulates HIF‐1 protein levels. (A) Western blot analysis of HIF‐1::MYC protein levels in *elc‐1* RNAi‐treated *C. elegans*. Knockdown of *elc‐1* increased HIF‐1::MYC protein levels. (B) Quantification of band intensity for data in panel A (*n* = 3). (C) Knockdown of *elc‐1* did not affect *hif‐1* mRNA levels in wild‐type or in *hif‐1* mutants (*n* = 4). (D–F) The effects of *elc‐1* RNAi on mRNA levels of the known HIF‐1 target genes, *nhr‐57* (D), *fmo‐2* (E), and *phy‐2* (F). *elc‐1* RNAi increased the mRNA levels of these genes in a *hif‐1*‐dependent manner (*n* = 4). An empty vector (L4440) was used as a negative control (Control RNAi). *vhl‐1* RNAi upregulates HIF‐1 and was used as a positive control. Error bars represent SEM (*P <* *0.05, **P <* *0.01, ***P <* *0.001, two‐tailed Student's *t*‐test).

Next, we determined whether ELC‐1 regulated HIF‐1 levels in *C. elegans*. Knockdown of *elc‐1* increased HIF‐1::MYC protein levels (Fig. 4A,B). However, *elc‐1* RNAi did not affect mRNA levels of *hif‐1* in quantitative RT–PCR (qRT–PCR) results (Fig. 4C). Thus, ELC‐1 affected HIF‐1 at the posttranscriptional level. We measured the expression levels of three *hif‐1* target genes, *nhr‐57*,* fmo‐2,* and *phy‐2* (Shen *et al*., 2005). We found that *elc‐1* RNAi upregulated mRNA levels of these genes in a *hif‐1*‐dependent manner (Fig. 4D–F). The effects of *elc‐1* RNAi were comparable to those of *vhl‐1* RNAi, which was used as a positive control (Fig. 4D–F). Thus, we concluded that *C. elegans* ELC‐1 negatively regulates HIF‐1 at the protein level, and this consistent with its function as an E3 ligase component in mammals.

**Figure 5:**
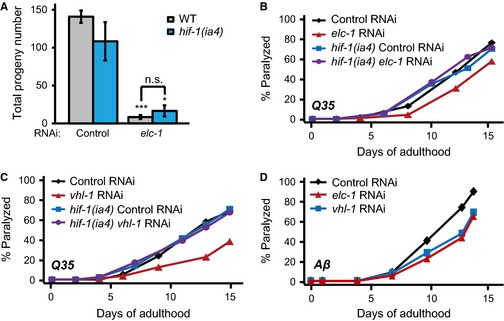
The effects of *elc‐1* RNAi on reproduction and protein homeostasis. (A) *elc‐1* RNAi caused severe sterility, which was not affected by *hif‐1(ia4)* mutations. (B, C) RNAi knockdown of *elc‐1* (four of five trials) (B) or *vhl‐1* (C) delayed the paralysis of transgenic worms expressing *Q35::YFP* (*Q35*). Delayed paralysis by *elc‐1* RNAi or *vhl‐1* RNAi was suppressed by *hif‐1* mutations. (D) *elc‐1* RNAi delayed the age‐dependent paralysis of Aβ (*A*β)‐expressing worms (two of three trials), similar to *vhl‐1* RNAi. See Table S3 for statistical analysis. Error bars represent SEM (n.s.: not significant, *P <* *0.05, ***P <* *0.001, two‐tailed Student's *t*‐test).

We examined whether knockdown of *elc‐1* affected other phenotypes, including impaired reproduction and improved protein homeostasis, which are caused by upregulation of HIF‐1 (Mehta *et al*., 2009). We found that *elc‐1* RNAi conferred a severe sterile phenotype, which was mostly independent of *hif‐1* (Fig. 5A). Age‐dependent paralysis in a transgenic worm model of Huntington's disease caused by expression of aggregation‐prone Q35 was reduced by *elc‐1* knockdown (Fig. 5B). The effect of *elc‐1* RNAi was similar to that of *vhl‐1* RNAi (Fig. 5C and Mehta *et al*., 2009). Knockdown of *elc‐1* or *vhl‐1* did not improve the motility of Q35 transgenic animals in a *hif‐1*‐mutant background (Fig. 5B,C). In addition, similar to *vhl‐1* RNAi (Mehta *et al*., 2009), *elc‐1* RNAi also delayed the paralysis caused by overexpression of aggregation‐prone Aβ, a worm model of Alzheimer's disease (Fig. 5D; two of three trials). Together, these data suggest that inhibition of ELC‐1 reduces proteotoxicity via HIF‐1 but affects reproduction independently of HIF‐1.

## Discussion

In this study, we analyzed the lifespan‐regulatory roles of putative HIF‐1 regulators in *C. elegans*. We showed that the inhibition of *C. elegans* elongin C promoted longevity by upregulating HIF‐1. Inhibition of elongin C also delayed paralysis in a *C. elegans* model of Huntington's disease that expresses a polyglutamine protein, in a HIF‐1‐dependent manner. In contrast, elongin C affected reproduction independently of HIF‐1. Thus, we propose that ELC‐1 regulates different aspects of animal physiology through both HIF‐1‐dependent and HIF‐1‐independent mechanisms.

Longevity caused by knockdown of the putative HIF‐1 regulators in this study was independent of *hif‐1* with the exception of *elc‐1* knockdown. In addition, a majority of the strong *nhr‐57* inducer RNAi clones increased the *nhr‐57p::gfp* levels in a largely *hif‐1*‐independent fashion. These findings are consistent with those of our previous report; RNAi of several mitochondrial ETC components increases the level of *nhr‐57p::gfp* and lifespan, but longevity is only marginally suppressed by *hif‐1* mutations (Lee *et al*., 2010). Thus, factors other than HIF‐1 appear to contribute to the regulation of *nhr‐57* expression and longevity. These factors may include transcription factors, DVE‐1, CEH‐23, and CEP‐1, which regulate longevity in response to inhibition of mitochondrial components (Durieux *et al*., 2011; Walter *et al*., 2011; Baruah *et al*., 2014), because *nhr‐57* is induced by impaired mitochondrial functions (Lee *et al*., 2010). In addition, transcription factors, such as ELT‐3, EOR‐1, BLMP‐1, ALR‐1, PHA‐4, PQM‐1, SKN‐1, MDL‐1, and PES‐1, bind to the promoter region of *nhr‐57*, based on modENCODE data analysis (Gerstein *et al*., 2010; Van Nostrand & Kim, 2013). It would be interesting to determine whether these transcription factors cooperate with HIF‐1 to regulate *nhr‐57* induction and longevity.

*C. elegans* has served as an excellent animal model for studying HIF‐1 biology, in particular due to a variety of available genetic tools, including viable *hif‐1* and its regulator mutants (Reviewed in Powell‐Coffman, 2010; Hwang & Lee, 2011). In addition, many studies have employed biochemical methods to measure the protein levels of HIF‐1 (Powell‐Coffman, 2010; Hwang & Lee, 2011; Romney *et al*., 2011; Ackerman & Gems, 2012; Hwang *et al*., 2014; this study). To our knowledge, however, no report has shown the ubiquitination patterns of HIF‐1 using *C. elegans*. Thus, it will be crucial for future research to determine the ubiquitination patterns of HIF‐1 to mechanistically dissect the E3 ligase functions of ELC‐1 and VHL‐1.

Genetic inhibition of VHL‐1 and ELC‐1 exerted similar and distinct effects on *C. elegans* physiology. Both *elc‐1* RNAi and *vhl‐1* mutation reduced fertility (shown in this study and in Mehta *et al*., 2009). However, *elc‐1* RNAi compromised reproduction independently of HIF‐1 (this study), whereas *vhl‐1* mutations do so in a HIF‐1‐dependent manner (Mehta *et al*., 2009). These differences may be due to the different roles of ELC‐1 and VHL‐1 in the E3 ligase complex. ELC‐1 is a core factor of E3 ligase and binds to multiple substrate‐specific subunits, including VHL‐1, which has limited specificity for the degradation of substrate proteins. Thus, it seems highly likely that ELC‐1 affects the stability of a broader range of substrates than VHL‐1 does. Indeed, an ELC‐1‐containing E3 ligase that contains ZIF‐1 as a substrate‐specific subunit affects reproduction by destabilizing PIE‐1, an essential factor for germ line development (DeRenzo *et al*., 2003). Thus, the reduced fertility caused by ELC‐1 may act through both the VHL‐1/HIF‐1 and ZIF‐1/PIE‐1 axes.

Studies have shown that elongin C has dual functions as an E3 ubiquitin ligase component and a transcription elongation factor (reviewed in Kim & Kaelin, 2003; Shilatifard *et al*., 2003). Nevertheless, the postembryonic functions of metazoan elongin C remain unclear. This is mainly due to the lethal phenotype of *Drosophila* (Mummery‐Widmer *et al*., 2009) and *C. elegans* (http://www.wormbase.org/species/c_elegans/gene/elc-1) mutants and the lack of a knockout mouse model. Here, we showed that elongin C modulated several physiologic aspects, including aging and reproduction. Thus, our work will serve as a guide for future research by improving our understanding of how elongin C regulates specific physiologic processes *in vivo*.

## Experimental procedures

### Strains

The following strains, including strains provided by Caenorhabditis Genetics Center, which is funded by the NIH National Center for Research Resources, were examined in this study: N2 wild‐type, ZG120 *iaIs7[nhr‐57p::gfp; unc‐119(+)]* a gift from Powell‐Coffman laboratory, IJ3 *hif‐1(ia4) V; iaIs7[nhr‐57p::gfp; unc‐119(+)]*, IJ6 *hif‐1(ia4) V* obtained by outcrossing ZG31 3 times to Lee laboratory N2, IJ159 *iaIs28[hif‐1p::hif‐1a::myc; unc‐119(+)]* obtained by outcrossing ZG580 4 times to Lee laboratory N2, IJ577 *yhEx136[elc‐1p::elc‐1::gfp; odr‐1p::rfp],* AM140 *rmIs132[unc‐54p::Q35::yfp],* CL2006 *dvls2[unc‐54p::Aβ 1‐42; rol‐6D]*, and IJ674 *hif‐1(ia4) V; rmIs132[unc‐54p::Q35::yfp]*.

### Examination of *nhr‐57p::gfp* expression upon RNAi treatment

RNAi bacteria were seeded onto the wells of 24‐well NGM plates in triplicate, and dsRNA was induced with 1 mm isopropyl‐β‐D‐thiogalactopyranoside (IPTG, Gold biotechnology, St. Louis, MO, USA) at room temperature for 24 h. *nhr‐57p::gfp* and *hif‐1(ia4); nhr‐57p::gfp* transgenic worms were synchronized on the RNAi bacteria lawns, and GFP expression was scored by three researchers in three independent experimental sets. GFP expression was scored as zero to three based on the intensity of GFP fluorescence. Zero indicated no induction, while three indicated the highest induction of GFP upon RNAi treatment. We set the criteria for the GFP scores using control RNAi (score: zero)‐ and *egl‐9* RNAi (score: three)‐treated *nhr‐57p::gfp*. Among 53 strong candidates, RNAi targeting 16 genes increased the *nhr‐57p::gfp* levels (arbitrary cutoff value = 0.5) in the solid culture system (Fig. 1A).

### Lifespan assays

Lifespan assays were performed as described previously with some modifications (Seo *et al*., 2013). Briefly, synchronized young adult worms were transferred onto 5 μm 5‐fluoro‐2′‐deoxyuridine (FUdR, Sigma, St. Louis, MO, USA)‐treated NGM plates with *E. coli* food. In the case of lifespan assays without FUdR treatment, worms were transferred to a new plate every one or 2 days until they stopped laying eggs. Approximately 100 worms for each condition were examined for death every 2 or 3 days until all the animals were dead. Animals that ruptured, bagged, burrowed, or crawled off the plates were censored but used as censored subjects for the statistical analysis. Lifespan assays were performed at 20°C. OASIS (http://sbi.postech.ac.kr/oasis) was used for statistical analysis (Yang *et al*., 2011).

### Analysis of the functional protein association networks

Functional protein association networks of genes identified from the genomewide RNAi screen (Lee *et al*., 2010) were analyzed using STRING (http://string-db.org/) (Franceschini *et al*., 2013). Networks were modified and revisualized using Cytoscape (http://www.cytoscape.org/) (Smoot *et al*., 2011).

### Modeling and alignment of protein structures

Modeling of *C. elegans* ELC‐1 and human TCEB1, a homolog of elongin C, was performed by using SWISS‐MODEL (http://swissmodel.expasy.org/) (Biasini *et al*., 2014), based on the crystal structures of mammalian VHL–elongin C–elongin B complex and SOCS3–elongin B–elongin C complex (Stebbins *et al*., 1999; Babon *et al*., 2008). Structures were revisualized and aligned using PyMOL (http://www.pymol.org/) (Schrodinger, 2010).

### Generation of plasmids for RNA interference and transgenesis

Genomic regions of *elc‐1,* Y82E9BR.3, and Y82E9BR.16 were cloned into pPD129.36 (Timmons *et al*., 2001) using In‐Fusion HD Cloning Kits (Clontech Laboratories, Inc., Mountain View, CA, USA), by following the manufacturer's instruction. Constructs were then transformed into HT115 competent cells. Genomic regions (approximately 3.5 kb, Fig. S2) of *elc‐1p::elc‐1* were cloned into pPD95.75 (Timmons *et al*., 2001) using HindIII and Acc65I restriction enzymes. Approximately 2 kb upstream sequences of *elc‐1* coding region were used as a promoter of *elc‐1*. Sequences of oligonucleotides used for generating the RNAi clones and the *elc‐1p::elc‐1::gfp* plasmid are as follows.

*elc‐1*‐F‐TCCACCGGTTCCATGGGCAAAAGTGGCCCAAAATCCGCT

*elc‐1*‐R‐GGGCGAATTGGGTACCACTAAAAATTCTGGGTTTCGTCATG

Y82E9BR.3‐F‐GAATTCGATATCAAGCTATTTTCAGGTAAAGAACTTTC

Y82E9BR.3‐R‐CTATAGGGCGAATTGGGATCCTTTTTTTCTCTCGTTCTCAG

Y82E9BR.16‐F‐GTGGATCCCCCGGGCATGAAGTTTGACGATTTCCTGTT

Y82E9BR.16‐R‐CTATAGGGCGAATTGGTTATGCGGTTTCTTCAGTGCTTTTCGAAAGAGGTTGC

*elc‐1p::elc‐1*‐F‐CCGGCAAGCTTATTGAAATTAAATAGAAAAAATTTG

*elc‐1p::elc‐1*‐R‐CAGTCGGTACCGAACAATCCAAGAAA

### Generation of *elc‐1p::elc‐1::gfp* transgenic animals

*elc‐1p::elc‐1::gfp* transgenic animals were generated as described previously with some modifications (Gaglia *et al*., 2012). The *elc‐1p::elc‐1::gfp* construct (25 ng μL^−1^) was injected into the gonad of day 1 adult hermaphrodites with a co‐injection marker, *odr‐1p::rfp* (75 ng μL^−1^).

### Quantification of paralysis

The quantification of paralyzed worms was performed as described previously (Mehta *et al*., 2009) with some modifications. Briefly, the paralysis of Q35 (Q35::YFP)‐ and Aβ‐expressing animals was determined by visual analysis. Worms were classified as paralyzed if they did not show any forward movement in response to tapping. Approximately 100 worms for each condition were examined for paralysis every 3 or 4 days until day 14 to day 16. Animals that died, ruptured, bagged, burrowed, or crawled off were censored but used for statistical analysis as censored subjects. All the paralysis assays were performed at 20°C. OASIS (http://sbi.postech.ac.kr/oasis) was used for statistical analysis (Yang *et al*., 2011). The format of Table S3 (Supporting information) that shows the statistical analysis of the paralysis experiments was based on a previous report (Zhang *et al*., 2013).

### Measurement of brood size

RNAi‐treated single L4 stage worm was placed on each RNAi bacteria‐seeded plate. Worms were transferred to new plates every day until they stopped laying eggs. The number of hatched progeny was counted. Six to nine P0 hermaphrodites were used for measuring average brood sizes.

### Western blot analysis

Synchronized young adult worms were harvested and washed using M9 buffer and then centrifuged at 2000 g for 5‐10 seconds. More than 1000 worms (approximately 50 μL of worm pellets) for each condition were used for one set of sample. Worms were then immediately frozen at −80°C and mixed with 2× SDS sample buffer. The samples were boiled at 100°C for 10 min and were vortexed until the samples were broken. After 30‐min centrifugation at 15 000 g, supernatant was used for the assay. The worm lysates were electrophoresed using 8% SDS‐PAGE and transferred to PVDF membrane. The membrane was treated with 5% skim milk for blocking and subsequently incubated with primary antibodies against c‐Myc (Santa Cruz, Paso Robles, CA, USA; 1:1000) or α‐tubulin (Santa Cruz, 1:1000). The membrane was then incubated with goat anti‐mouse secondary antibody conjugated with horseradish peroxidase (Thermo, Waltham, MA, USA, 1:10 000). The PVDF membrane was then treated with the chemiluminescent horseradish peroxidase substrate (Thermo) for 1 min, and the signal was detected using X‐ray film (Agfa, Mortsel, Belgium). The band intensity was quantified using imagej (http://imagej.nih.gov/ij/).

### Fluorescence microscopy

Images of *elc‐1p::elc‐1::gfp* animals were taken using an AxioCam HRc CCD digital camera (Zeiss Corporation, Jena, Germany) with a Zeiss Axio Scope A1 compound microscope (Zeiss Corporation). Tetramisole hydrochloride (0.4 mm; Sigma) was used as an anesthetic.

### Quantitative RT–PCR

Approximately 500–1000 RNAi‐treated young adult worms were used for the quantitative RT–PCR analysis. Preparation of cDNA samples was performed as previously described (Lee *et al*., 2010). Quantitative PCR from the cDNA was executed in a StepOne Real Time PCR System (Applied Biosystems, Foster City, CA, USA) and analyzed using comparative C_T_ method. mRNA levels of *ama‐1* (the large subunit of RNA polymerase II) were used for normalization.

### List of oligonucleotides used for the quantitative RT–PCR

*ama‐1*‐F‐TGGAACTCTGGAGTCACACC
*ama‐1*‐R‐CATCCTCCTTCATTGAACGG
*hif‐1*‐F‐CAGTGATTCTTCAATTCTTTACGTC
*hif‐1*‐R‐GGATTAACACAGACAGATTTAACAG
*nhr‐57*‐F‐GACTCTGTGTGGAGTGATGGAGAG
*nhr‐57*‐R‐GTGGCTCTTGGTGTCAATTTCGGG
*fmo‐2‐*F‐GTCACTTGTTTTGAGGCGTCAGATG
*fmo‐2*‐R‐CATAACTGACGACTCATTCGTTTCG
*phy‐2*‐F‐GTATGAGGACATGCTTCAAGGAAAG
*phy‐2*‐R‐CATCGTACTCAACTCTCTTGTTAAC
*elc‐1‐*F‐GGGAGCTCGCGCTTACCTCTGGAAC
*elc‐1‐*R‐GTAGACGACGTTGCTCTCGTTCTCG
Y82E9BR.3‐F‐CCTCCTCGCCTCGAGAGCCCCACTC
Y82E9BR.3‐R‐CGGCTCCAGCTCCGATGTACTTGGC

## Funding

This work was supported by the Korean Health Technology R&D Project, Ministry of Health & Welfare, Republic of Korea (HI11C1609 and HI14C2337), to S.‐J.V.L.

## Conflict of interest

The authors declare no conflict of interests.

## Supporting information


**Fig. S1** Network analysis of putative negative regulators of HIF‐1.
**Fig. S2** Graphical information of *elc‐1p::elc‐1* and RNAi clones against *elc‐1*, Y82E9BR.3, and Y82E9BR.16.
**Fig. S3** Lifespan results of wild‐type animals treated with *nhr‐57* inducer RNAi clones, which did not extend lifespan.
**Fig. S4** Lifespan results of *hif‐1* mutants treated with *nhr‐57* inducer RNAi clones, which increased the lifespan of wild‐type animals.
**Fig. S5** Dissection of the effects of the *elc‐1*, Y82E9BR.3, and Y82E9BR.16 RNAi clones.

**Table S1** The list of RNAi clones that highly increased the level of *nhr‐57p::gfp* in a liquid culture system.

**Table S2** Analysis of lifespan assay results.
**Table S3** Analysis of paralysis assay results.
