# Supplementary figures and images for: Inhibition of elongin C promotes longevity and protein homeostasis via HIF‐1 in C. elegans
Source: Aging Cell. 2015 Sep 11;14(6):995–1002. doi: 10.1111/acel.12390 (PMC4693473; doi:10.1111/acel.12390)

Figure S1

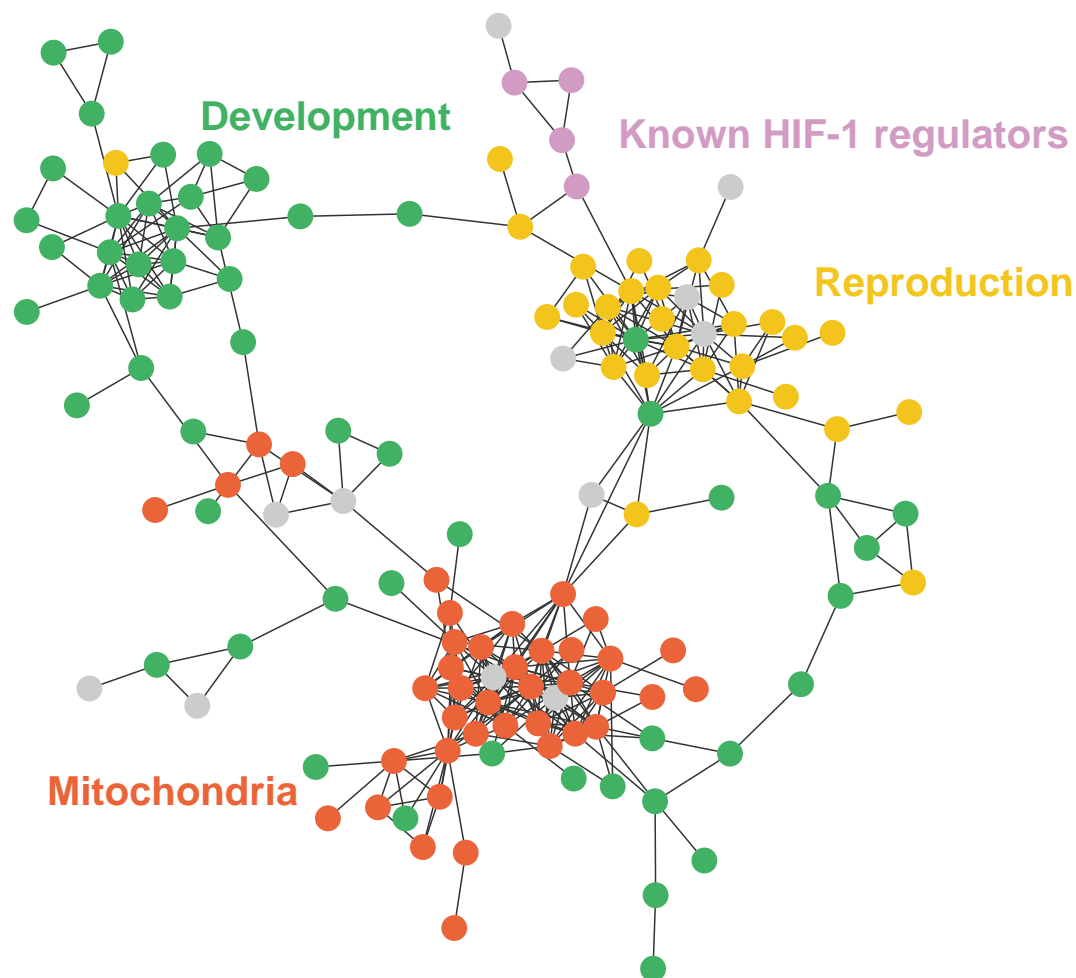

Figure S2

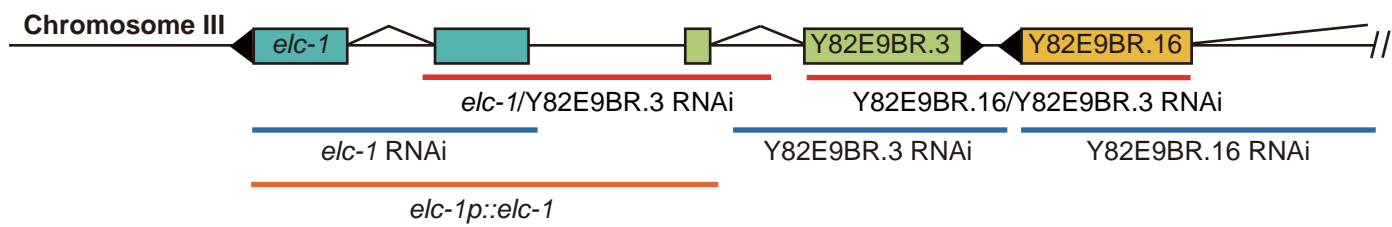

**Figure S3**

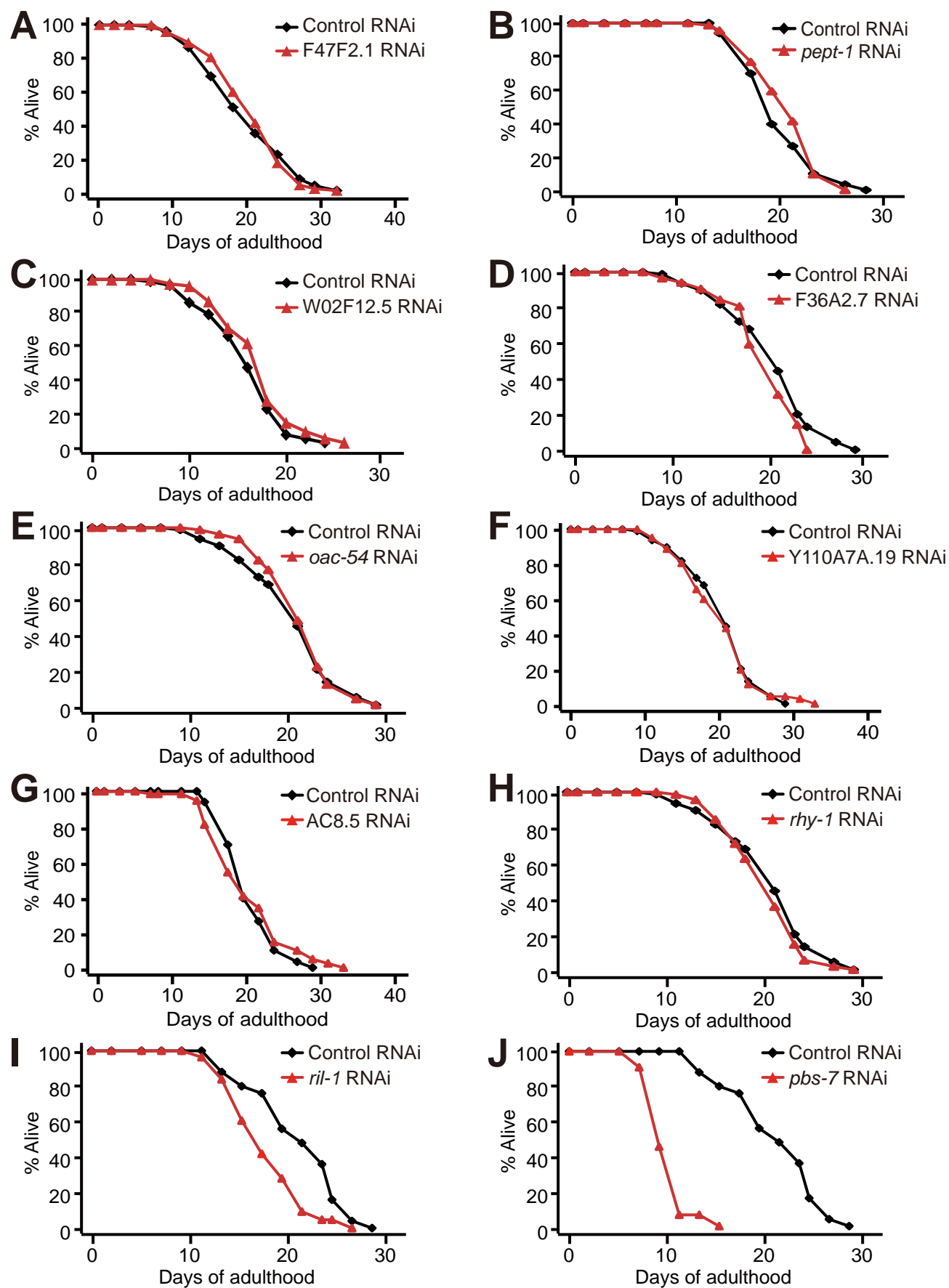

**Figure S4**

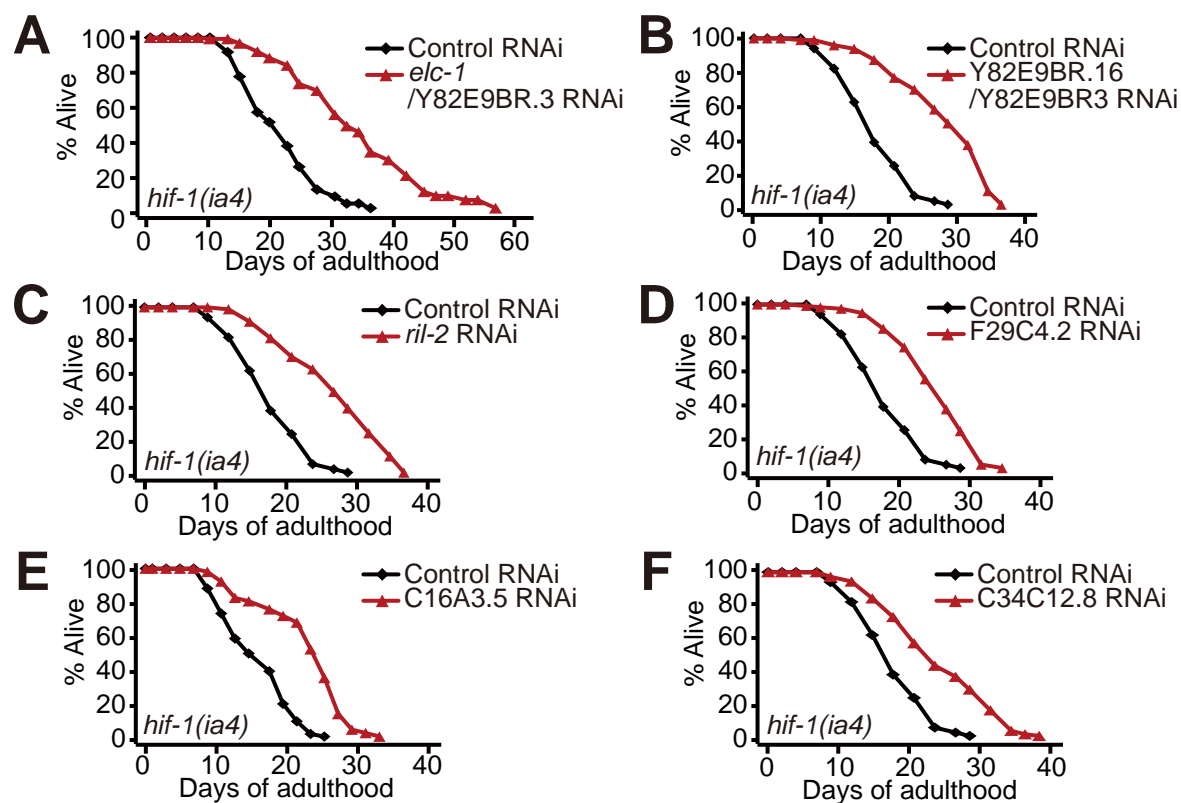

# Figure S5

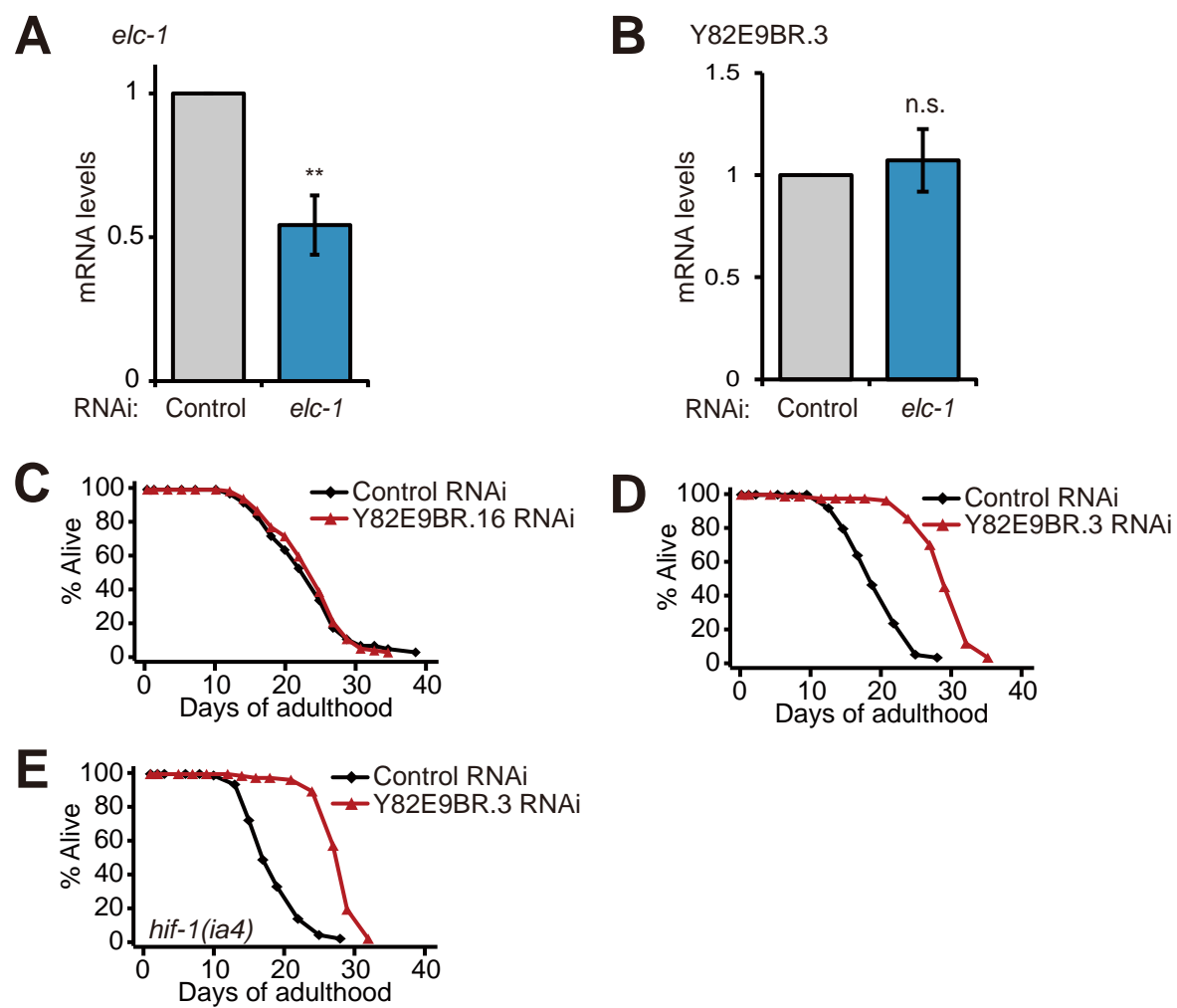

Supplement: Supplementary file 1 — Fig. S1 Network analysis of putative negative regulators of HIF‐1. Fig. S2 Graphical information of elc‐1p::elc‐1 and RNAi clones against elc‐1, Y82E9BR.3, and Y82E9BR.16. Fig. S3 Lifespan results of wild‐type animals treated with nhr‐57 inducer RNAi clones, which did not extend lifespan. Fig. S4 Lifespan results of hif‐1 mutants treated with nhr‐57 inducer RNAi clones, which increased the lifespan of wild‐type animals. Fig. S5 Dissection of the effects of the elc‐1, Y82E9BR.3, and Y82E9BR.16 RNAi clones. [file ACEL-14-0995-s001.pdf]
