# Supplementary material for: Inhibition of elongin C promotes longevity and protein homeostasis via HIF‐1 in C. elegans
Source: Aging Cell. 2015 Sep 11;14(6):995–1002. doi: 10.1111/acel.12390 (PMC4693473; doi:10.1111/acel.12390)
Supplement: Supplementary file 3 — Table S2 Analysis of lifespan assay results. Table S3 Analysis of paralysis assay results. [file ACEL-14-0995-s003.docx]

**Supplementary Information**

**Supplementary Figure Legends**

**Figure S1. Network analysis of putative negative regulators of HIF-1.**

Functional protein association networks of putative negative regulators of HIF-1 identified from our previous work (Lee *et al.,* 2010). We found four major functional groups. As we previously reported (Lee *et al.,* 2010), one group consists of genes that encode mitochondrial proteins. We found additional clusters for genes involved in development and reproduction, and a cluster of known HIF-1 regulators.

**Figure S2. Graphical information of *elc-1p::elc-1* and RNAi clones against *elc-1*, Y82E9BR.3, and Y82E9BR.16.**

The orange bar indicates the genomic region of *elc-1p::elc-1*, which was used for transgenesis. We found that these *elc-1*/Y82E9BR.3 and Y82E9BR.16/Y82E9BR.3 RNAi clones shared a common target gene, Y82E9BR.3 (worm homolog of ATP synthase subunit c) as marked with red bars. We therefore constructed RNAi clones that individually targeted *elc-1*, Y82E9BR.3, and Y82E9BR.16 (indicated in blue bars).

**Figure S3.** **Lifespan results of wild-type animals treated with *nhr-57* inducer RNAi clones, which did not extend lifespan.** Lifespan curves of worms treated with RNAi targeting F47F2.1 (**A**), *pept-1* (**B**), W02F12.5 (**C**), F36A2.7 (**D**), *oac-54* (**E**), Y110A7A.19 (**F**), AC8.5 (**G**), *rhy-1* (**H**), *ril-1* (**I**), and *pbs-7* (**J**). Lifespan assays were performed at least twice independently. See Table S2 for additional trials and statistical analysis. Please note that knockdown of F47F2.1 or *pept-1* increased the lifespan of wild-type animals in some trials (2 out of 4 trials and 1 out of 2 trials, respectively, Table S2). As the results were variable, we conservatively show negative data in panels **A** and **B**.

**Figure S4.** **Lifespan results of *hif-1* mutants treated with *nhr-57* inducer RNAi clones, which increased the lifespan of wild-type animals.** Lifespan curves of *hif-1(ia4)* mutants treated with RNAi targeting *elc-1*/Y82E9BR.3 (**A**), Y82E9BR.16/Y82E9BR.3 (**B**), *ril-2* (**C**), F29C4.2 (**D**), C16A3.5 (**E**), and C34C12.8 (**F**). Lifespan assays were performed at least twice independently. See Table S2 for additional trials and statistical analysis.

**Figure S5. Dissection of the effects of the *elc-1*, Y82E9BR.3, and Y82E9BR.16 RNAi clones.** We noticed that the commercially available RNAi clone targeting *elc-1*/Y82E9BR.3 or Y82E9BR.16/Y82E9BR.3 elicited a robust (greater than 50%: Fig. 1B and C) increase in lifespan. We therefore dissected the effects of *elc-1*, Y82E9BR.3, and Y82E9BR.16 RNAi on lifespan (Fig. S2). We found that *elc-1* RNAi promoted longevity in a *hif-1*-dependent manner (Fig. 2A and B). (**A** and **B**) We confirmed that this in-house *elc-1* RNAi specifically decreased the mRNA level of *elc-1* (n=6) (**A**) but not that of Y82E9BR.3 (n=3) (**B**) by using qRT-PCR. (**C**) Y82E9BR.16 RNAi did not increase the lifespan of wild-type. (**D** and **E**) Y82E9BR.3 RNAi extended the lifespan of both wild-type (**D**) and *hif-1(ia4)* mutant animals (**E**). Error bars represent s.e.m (n.s.: not significant, ***p*<0.01, two-tailed Student’s t-test). See Table 1 and Table S2 for additional information for lifespan data shown in this figure.

**Table S1.** **The list of RNAi clones that highly increased the level of *nhr-57p::gfp* in a liquid culture system.** Please see the separate spreadsheet file.

**Table S2. Analysis of lifespan assay results.**

| **Strain** | **Mean lifespan ±s.e.m. (days)** | **75th percentile** | **% change** | **Number of animals that died/total** | ***p* value vs. control** | **Figure in text** |
| --- | --- | --- | --- | --- | --- | --- |
| WT control | 20.6±0.5 | 23 |  | 72/85 |  | Fig. S3D-F, S3H |
| *egl-9(RNAi)* | 23.8±0.5 | 27 | +15% | 63/89 | 0.0001 |  |
| *rhy-1(RNAi)* | 20.2±0.5 | 23 | -2% | 59/82 | 0.2752 | Fig. S3H |
| *oac-54(RNAi)* | 21.5±0.4 | 24 | +4% | 71/89 | 0.5797 | Fig. S3E |
| Y110A7A.19*(RNAi)* | 20.3±0.6 | 23 | -1% | 73/87 | 0.9369 | Fig. S3F |
| F36A2.7*(RNAi)* | 20.8±0.4 | 23 | +1% | 74/89 | 0.3173 | Fig. S3D |
| F29C4.2*(RNAi)* | 32.2±0.6 | 36 | +56% | 65/80 | <0.0001 |  |
| W02F12.5*(RNAi)* | 23.0±0.9 | 28 | +12% | 62/78 | 0.0004 |  |
| *ril-2(RNAi)* | 30.9±0.7 | 36 | +50% | 66/85 | <0.0001 |  |
| *elc-1/Y82E9BR.3(RNAi)* | 29.5±0.6 | 34 | +43% | 71/90 | <0.0001 |  |
| C34C12.8*(RNAi)* | 23.3±0.9 | 28 | +17% | 43/71 | 0.0018 |  |
| C16A3.5*(RNAi)* | 24.0±0.6 | 27 | +21% | 49/76 | <0.0001 |  |
| F47F2.1*(RNAi)* | 26.2±0.6 | 31 | +31% | 60/75 | <0.0001 |  |
| Y82E9BR.16/Y82E9BR.3*(RNAi)* | 25.4±0.6 | 28 | +28% | 46/84 | <0.0001 |  |
| WT control | 16.0±0.4 | 18 |  | 83/90 |  | Fig. S3C |
| *egl-9(RNAi)* | 17.3±0.3 | 20 | +9% | 82/90 | 0.1066 |  |
| *rhy-1(RNAi)* | 15.2±0.3 | 18 | -5% | 70/90 | 0.0212 |  |
| *oac-54(RNAi)* | 16.4±0.3 | 20 | +2% | 80/90 | 0.5052 |  |
| Y110A7A.19*(RNAi)* | 16.6±0.4 | 20 | +4% | 78/90 | 0.2490 |  |
| F36A2.7*(RNAi)* | 17.3±0.3 | 20 | +9% | 85/90 | 0.0351 |  |
| F29C4.2*(RNAi)* | 24.4±0.7 | 28 | +53% | 50/60 | <0.0001 |  |
| W02F12.5*(RNAi)* | 17.2±0.4 | 20 | +7% | 74/90 | 0.0516 | Fig. S3C |
| *ril-2(RNAi)* | 22.8±0.6 | 28 | +43% | 80/90 | <0.0001 |  |
| *elc-1/Y82E9BR.3(RNAi)* | 21.1±0.5 | 24 | +32% | 81/90 | <0.0001 |  |
| WT control | 14.2±0.5 | 16 |  | 36/84 |  |  |
| *egl-9(RNAi)* | 17.2±0.4 | 20 | +21% | 73/88 | <0.0001 |  |
| *pept-1(RNAi)* | 17.6±0.5 | 20 | +23% | 61/86 | <0.0001 |  |
| AC8.5*(RNAi)* | 14.7±0.3 | 18 | +3% | 60/83 | 0.0756 |  |
| C34C12.8*(RNAi)* | 15.4±0.7 | 20 | +8% | 60/71 | 0.0069 |  |
| C16A3.5*(RNAi)* | 19.6±0.8 | 26 | +38% | 49/80 | <0.0001 |  |
| F47F2.1*(RNAi)* | 20.0±0.7 | 24 | +41% | 52/76 | <0.0001 |  |
| Y82E9BR.16/Y82E9BR.3*(RNAi)* | 21.8±0.6 | 24 | +53% | 58/82 | <0.0001 |  |
| WT control | 19.9±0.6 | 23 |  | 32/78 |  | Fig. S3B, S3G |
| *egl-9(RNAi)* | 24.6±0.5 | 28 | +24% | 54/73 | <0.0001 |  |
| *pept-1(RNAi)* | 20.7±0.4 | 23 | +4% | 58/82 | 0.2921 | Fig. S3B |
| AC8.5*(RNAi)* | 19.6±0.7 | 23 | -1% | 44/80 | 0.9261 | Fig. S3G |
| WT control^α^ | 20.7±0.9 | 24 |  | 25/82 |  | Fig. S3I and J |
| *egl-9(RNAi)*^α^ | 22.3±0.6 | 24 | +7% | 53/80 | 0.1143 |  |
| *pbs-7(RNAi)*^α^ | 10.0±0.4 | 13 | -52% | 19/83 | <0.0001 | Fig. S3J |
| *ril-1(RNAi)*^α^ | 17.5±0.6 | 21 | -15% | 22/89 | 0.0064 | Fig. S3I |
| WT control^α^ | 16.0±0.7 | 20 |  | 57/85 |  |  |
| *egl-9(RNAi)*^α^ | 18.2±0.4 | 20 | +14% | 70/85 | 0.1042 |  |
| *pbs-7(RNAi)*^α^ | 8.0±0.2 | 13 | -50% | 47/85 | <0.0001 |  |
| *ril-1(RNAi)*^α^ | 12.1±0.4 | 14 | -24% | 46/84 | <0.0001 |  |
| WT control | 20.0±0.6 | 24 |  | 102/120 |  | Fig. 1C-E, 1G, and S3A |
| *egl-9(RNAi)* | 20.1±0.6 | 24 | +1% | 79/120 | 1.0000 |  |
| F29C4.2*(RNAi)* | 25.0±0.6 | 29 | +25% | 108/120 | <0.0001 | Fig. 1E |
| *ril-2(RNAi)* | 27.2±0.6 | 32 | +36% | 98/120 | <0.0001 | Fig. 1D |
| C34C12.8*(RNAi)* | 24.1±0.7 | 29 | +21% | 101/120 | <0.0001 | Fig. 1G |
| C16A3.5*(RNAi)* | 22.9±0.6 | 27 | +15% | 91/115 | 0.0164 |  |
| F47F2.1*(RNAi)* | 20.6±0.5 | 24 | +3% | 91/120 | 1.0000 | Fig. S3A |
| Y82E9BR.16/Y82E9BR.3*(RNAi)* | 29.4±0.6 | 27 | +47% | 95/120 | <0.0001 | Fig. 1C |
| *hif-1(ia4)* control | 18.1±0.5 | 21 | -9% | 99/120 | 0.0868 | Fig. S4B-D, S4F |
| *egl-9(RNAi) hif-1(ia4)* | 19.1±0.5 | 24 | +5% | 82/120 | 1.0000  (1.0000^WT^ *^elg-9^*) |  |
| F29C4.2*(RNAi); hif-1(ia4)* | 25.6±0.5 | 29 | +41% | 101/120 | <0.0001  (1.0000^WT F29C4.2^) | Fig. S4D |
| *ril-2(RNAi) hif-1(ia4)* | 27.2±0.6 | 32 | +50% | 78/85 | <0.0001  (1.0000^WT^ *^ril-2^*) | Fig. S4C |
| C34C12.8*(RNAi); hif-1(ia4)* | 24.6±0.7 | 32 | +35% | 103/115 | <0.0001  (1.0000^WT C34C12.8^) | Fig. S4F |
| C16A3.5*(RNAi); hif-1(ia4)* | 22.3±0.8 | 32 | +23% | 78/90 | <0.0001  (1.0000^WT C16A3.5^) |  |
| *hif-1(ia4);* F47F2.1*(RNAi)* | 19.5±0.5 | 24 | +8% | 78/120 | 1.0000  (0.9007^WT F47F2.1^) |  |
| Y82E9BR.16/Y82E9BR.3*(RNAi); hif-1(ia4)* | 28.5±0.8 | 35 | +57% | 77/105 | <0.0001  (1.0000^WT Y82E9BR.16/Y82E9BR.3^) | Fig. S4B |
| WT control | 18.4±0.7 | 24 |  | 78/120 |  | Fig. 1F |
| *egl-9(RNAi)* | 17.7±0.6 | 22 | -4% | 84/120 | 1.0000 |  |
| F29C4.2*(RNAi)* | 22.0±0.6 | 26 | +19% | 93/120 | 0.0067 |  |
| *ril-2(RNAi)* | 23.4±0.7 | 28 | +27% | 97/120 | <0.0001 |  |
| C34C12.8*(RNAi)* | 23.1±0.7 | 28 | +26% | 75/120 | <0.0001 |  |
| C16A3.5*(RNAi)* | 21.9±0.7 | 26 | +19% | 97/120 | 0.0026 | Fig. 1F |
| F47F2.1*(RNAi)* | 19.1±0.7 | 22 | +4% | 62/120 | 1.0000 |  |
| Y82E9BR.16/Y82E9BR.3*(RNAi)* | 26.8±0.6 | 32 | +45% | 86/120 | <0.0001 |  |
| *hif-1(ia4)* control | 16.3±0.6 | 20 | -12% | 67/120 | 0.0275 | Fig. S4E |
| *egl-9(RNAi) hif-1(ia4)* | 16.9±0.7 | 20 | +4% | 64/120 | 1.0000  (1.0000^WT^ *^elg-9^*) |  |
| F29C4.2*(RNAi); hif-1(ia4)* | 21.5±0.6 | 26 | +32% | 110/120 | <0.0001  (1.0000^WT F29C4.2^) |  |
| *ril-2(RNAi) hif-1(ia4)* | 26.6±0.7 | 30 | +64% | 91/120 | <0.0001  (0.0283^WT^ *^ril-2^*) |  |
| C34C12.8*(RNAi); hif-1(ia4)* | 23.4±0.8 | 28 | +44% | 57/90 | <0.0001  (1.0000^WT C34C12.8^) |  |
| C16A3.5*(RNAi); hif-1(ia4)* | 21.9±0.7 | 26 | +35% | 100/120 | <0.0001  (1.0000^WT C16A3.5^) | Fig. S4E |
| *hif-1(ia4);* F47F2.1*(RNAi)* | 15.7±0.7 | 20 | -3% | 56/120 | 1.0000  (0.0032^WT F47F2.1^) |  |
| Y82E9BR.16/Y82E9BR.3*(RNAi); hif-1(ia4)* | 28.2±0.6 | 32 | +73% | 107/120 | <0.0001  (0.6126^WT Y82E9BR.16/Y82E9BR.3^) |  |
| WT control | 22.3±0.6 | 27 |  | 93/125 |  | Fig. 2A |
| *elc-1(RNAi)* | 26.8±0.5 | 30 | +20% | 74/100 | <0.0001 | Fig. 2A |
| *hif-1(ia4)* control | 20.9±0.7 | 23 | -7% | 88/115 | 0.1049 |  |
| *elc-1(RNAi); hif-1(ia4)* | 23.4±0.5 | 26 | +12% | 88/125 | 0.0342 (<0.0001^WT^ *^elc-1^*) |  |
| WT control | 19.0±0.6 | 23 |  | 66/150 |  |  |
| *elc-1(RNAi)* | 21.9±0.4 | 25 | +15% | 104/151 | 0.0003 |  |
| *elc-1/Y82E9BR.3(RNAi)* | 34.7±0.8 | 42 | +83% | 114/150 | <0.0001 |  |
| *hif-1(ia4)* control | 21.0±0.6 | 25 | +10% | 101/151 | 0.0102 | Fig. 2B |
| *elc-1(RNAi); hif-1(ia4)* | 21.1±0.4 | 25 | 0% | 119/150 | 0.4000 (0.2028 ^WT^ *^elc-1^*) | Fig. 2B |
| WT control | 21.7±0.6 | 25 |  | 89/150 |  |  |
| *elc-1(RNAi)* | 22.9±0.6 | 28 | +5% | 97/150 | 0.1089 |  |
| *elc-1/Y82E9BR.3(RNAi)* | 37.8±0.6 | 46 | +74% | 83/125 | <0.0001 |  |
| *hif-1(ia4)* control | 21.8±0.6 | 25 | 0% | 91/125 | 0.7185 |  |
| *elc-1(RNAi); hif-1(ia4)* | 22.9±0.4 | 25 | +5% | 97/151 | 0.3804 (0.5036 ^WT^ *^elc-1^*) |  |
| *elc-1/Y82E9BR.3(RNAi); hif-1(ia4)* | 34.4±1.0 | 43 | +58% | 117/150 | <0.0001 (0.004^WT^ *^elc/^*^Y82E9BR.3^) |  |
| WT control | 21.8±0.5 | 26 |  | 82/150 |  |  |
| *elc-1(RNAi)* | 23.8±0.5 | 26 | +10% | 93/125 | 0.0063 |  |
| *hif-1(ia4)* control | 21.3±0.6 | 25 | -2% | 60/125 | 0.2357 |  |
| *elc-1(RNAi); hif-1(ia4)* | 20.0±0.5 | 23 | -6% | 74/125 | 0.0501 (<0.0001^WT^ *^elc-1^*) |  |
| WT control | 20.2±0.5 | 25 |  | 93/120 |  |  |
| *elc-1(RNAi)* | 22.5±0.5 | 25 | +11% | 88/120 | 0.0024 |  |
| *hif-1(ia4)* control | 21.0±0.6 | 27 | +4% | 85/120 | 0.3670 |  |
| *elc-1(RNAi); hif-1(ia4)* | 20.5±0.7 | 24 | -2% | 60/90 | 0.5416 (0.0024 ^WT^ *^elc-1^*) |  |
| WT control | 17.5±0.4 | 20 |  | 93/150 |  |  |
| *elc-1(RNAi)* | 19.8±0.5 | 22 | +13% | 114/150 | 0.0006 |  |
| *hif-1(ia4)* control | 16.0±0.3 | 18 | -9% | 131/150 | 0.0026 |  |
| *elc-1(RNAi); hif-1(ia4)* | 17.1±0.3 | 20 | +7% | 129/150 | 0.0087 (<0.0001^WT^ *^elc-1^*) |  |
| WT control | 21.7±0.5 | 27 |  | 104/150 |  |  |
| *elc-1(RNAi)* | 21.9±0.5 | 25 | +1% | 99/150 | 0.6586 |  |
| *hif-1(ia4)* control | 18.6±0.5 | 22 | -17% | 106/150 | 0.0001 |  |
| *elc-1(RNAi); hif-1(ia4)* | 19.2±0.4 | 22 | +3% | 59/150 | 0.6390 (<0.0001^WT^ *^elc-1^*) |  |
| WT control^#^ | 21.5±0.6 | 27 |  | 94/120 |  |  |
| *elc-1(RNAi)^#^* | 24.1±0.5 | 27 | +12% | 108/120 | 0.0001 |  |
| *hif-1(ia4)* control^#^ | 20.5±0.6 | 24 | -4% | 86/120 | 0.2975 |  |
| *elc-1(RNAi); hif-1(ia4)^#^* | 21.7±0.5 | 27 | +6% | 99/120 | 0.1825 (0.0005^WT^ *^elc-1^*) |  |
| WT control*^#^* | 22.7±0.5 | 26 |  | 89/120 |  |  |
| *elc-1(RNAi) ^#^* | 24.1±0.6 | 29 | +6% | 93/120 | 0.0144 |  |
| *hif-1(ia4)* control*^#^* | 20.9±0.7 | 26 | -9% | 83/120 | 0.5532 |  |
| *elc-1(RNAi); hif-1(ia4) ^#^* | 23.9±0.7 | 29 | +14% | 83/120 | 0.0070 (0.7279^WT^ *^elc-1^*) |  |
| WT control | 23.0±0.6 | 27 |  | 106/125 |  | Fig. S5D |
| Y82E9BR.16/Y82E9BR.3*(RNAi)* | 34.2±0.7 | 40 | +49% | 105/125 | <0.0001 |  |
| Y82E9BR.16*(RNAi)* | 23.7±0.5 | 27 | +3% | 101/125 | 0.5407 | Fig. S5D |
| WT control | 19.0±0.4 | 23 |  | 142/150 |  |  |
| Y82E9BR.16/Y82E9BR.3*(RNAi)* | 32.6±0.5 | 37 | +71% | 121/125 | <0.0001 |  |
| Y82E9BR.16*(RNAi)* | 18.8±0.4 | 21 | -1% | 145/150 | 0.7110 |  |
| WT control | 18.7±0.4 | 21 |  | 110/150 |  | Fig. S5E |
| Y82E9BR.3*(RNAi)* | 28.1±0.5 | 31 | +51% | 81/92 | <0.0001 | Fig. S5E |
| *hif-1(ia4)* control | 17.6±0.4 | 21 | -6% | 92/150 | 0.0481 | Fig. S5F |
| Y82E9BR.3*(RNAi); hif-1(ia4)* | 27.1±0.3 | 31 | +54% | 83/96 | <0.0001 (0.0010^WT Y82E9BR.3^) | Fig. S5F |
| WT control | 17.9±0.3 | 21 |  | 127/150 |  |  |
| Y82E9BR.3*(RNAi)* | 27.8±0.3 | 31 | +56% | 74/100 | <0.0001 |  |
| *hif-1(ia4)* control | 17.8±0.4 | 21 | -1% | 110/150 | 0.6953 |  |
| Y82E9BR.3*(RNAi); hif-1(ia4)* | 27.8±0.5 | 31 | +57% | 51/80 | <0.0001 (0.2052^WT Y82E9BR.3^) |  |
| WT control | 22.4±0.5 | 25 |  | 99/150 |  | Fig. 1B |
| *elc-1/Y82E9BR.3(RNAi)* | 28.4±0.6 | 33 | +27% | 102/150 | <0.0001 | Fig. 1B |
| *hif-1(ia4)* control | 24.6±0.6 | 27 | +9% | 71/125 | 0.0716 | Fig. S4A |
| *elc-1/Y82E9BR.3(RNAi); hif-1(ia4)* | 30.8±0.7 | 37 | +25% | 94/124 | <0.0001(0.0017^WT^ *^elc/^*^Y82E9BR.3^) | Fig. S4A |
| *hif-1(ia4)* control | 21.8±0.7 | 26 |  | 78/150 |  |  |
| *elc-1*/Y82E9BR.3*(RNAi); hif-1(ia4)* | 29.9±0.7 | 35 | +37% | 111/151 | <0.0001 |  |

In this table, the survival data sets within solid lines were done in parallel and statistical analysis was done within the sets. Differences in conditions were distinguished by dotted lines. *p* values were calculated within the sets, and were calculated by using the log-rank (Mantel-Cox) method. Percent changes of mean lifespan of control RNAi-treated mutants were calculated against those of control RNAi-treated wild-type worms. Percent changes of mean lifespan of RNAi-treated wild-type and mutant animals were calculated against control RNAi-treated wild-type and mutant worms, respectively. *p* values for each condition were calculated against the control immediately above.

^α^: lifespan assays with RNAi treatment only during adulthood.

^#^: lifespan assays without FUdR treatment.

**Table S3. Analysis of paralysis assay results.**

| **Strain** | **Mean motility (not paralyzed) ±s.e.m. (days)** | **% change** | **Number of animals that paralyzed/total** | ***p* value vs. control** | **Figure in text** |
| --- | --- | --- | --- | --- | --- |
| *Q35::YFP* control | 10.9±0.3 |  | 98/120 |  |  |
| *elc-1(RNAi); Q35::YFP* | 12.1±0.3 | +12% | 71/120 | 0.0094 |  |
| *vhl-1(RNAi); Q35::YFP* | 12.7±0.3 | +17% | 71/120 | <0.0001 |  |
| *hif-1(ia4); Q35::YFP* control | 13.6±0.3 | +20% | 68/120 | <0.0001 |  |
| *elc-1(RNAi); hif-1(ia4); Q35::YFP* | 12.4±0.4 | -9% | 86/120 | 0.0024 (0.7687 *^Q35 elc-1^*) |  |
| *hif-1(ia4); vhl-1(RNAi); Q35::YFP* | 13.3±0.3 | -2% | 70/120 | 0.1536 (0.8334 *^Q35 vhl-1^*) |  |
| *Q35::YFP* control | 11.4±0.3 |  | 98/120 |  |  |
| *elc-1(RNAi); Q35::YFP* | 12.9±0.3 | +13% | 87/120 | 0.0001 |  |
| *vhl-1(RNAi); Q35::YFP* | 13.2±0.3 | +16% | 81/120 | <0.0001 |  |
| *Q35::YFP* control | 12.1±0.4 |  | 54/90 |  |  |
| *elc-1(RNAi); Q35::YFP* | 13.5±0.3 | +11% | 75/120 | 0.0114 |  |
| *vhl-1(RNAi); Q35::YFP* | 13.8±0.3 | +14% | 63/120 | 0.0003 |  |
| *hif-1(ia4); Q35::YFP* control | 14.8±0.2 | +23% | 67/120 | <0.0001 |  |
| *elc-1(RNAi); hif-1(ia4); Q35::YFP* | 14.0±0.3 | -5% | 59/120 | 0.3024 (0.0173 *^Q35 elc-1^*) |  |
| *hif-1(ia4); vhl-1(RNAi); Q35::YFP* | 14.1±0.3 | -5% | 58/120 | 0.4180 (0.1904 *^Q35 vhl-1^*) |  |
| *Q35::YFP* control | 12.6±0.3 |  | 51/120 |  | Fig. 5C |
| *elc-1(RNAi); Q35::YFP* | 13.6±0.3 | +8% | 42/120 | 0.0219 |  |
| *vhl-1(RNAi); Q35::YFP* | 13.9±0.3 | +10% | 29/90 | <0.0001 | Fig. 5C |
| *hif-1(ia4); Q35::YFP* control | 12.3±0.3 | -2% | 84/120 | 0.5389 | Fig. 5C |
| *elc-1(RNAi); hif-1(ia4); Q35::YFP* | 11.4±0.3 | -8% | 86/120 | 0.1752 (0.0001 *^Q35 elc-1^*) |  |
| *hif-1(ia4); vhl-1(RNAi); Q35::YFP* | 12.3±0.3 | 0% | 77/120 | 0.6689 (<0.0001*^Q35^* *^vhl-1^*) | Fig. 5C |
| *Q35::YFP* control | 12.8±0.3 |  | 67/120 |  | Fig. 5B |
| *elc-1(RNAi); Q35::YFP* | 13.7±0.2 | +8% | 46/120 | 0.0009 | Fig. 5B |
| *vhl-1(RNAi); Q35::YFP* | 14.1±0.2 | +10% | 28/120 | <0.0001 |  |
| *hif-1(ia4); Q35::YFP* control | 12.4±0.3 | -3% | 85/120 | 0.8733 | Fig. 5B |
| *elc-1(RNAi); hif-1(ia4); Q35::YFP* | 12.0±0.3 | -3% | 82/120 | 0.3247 (0.0004 *^Q35 elc-1^*) | Fig. 5B |
| *hif-1(ia4); vhl-1(RNAi); Q35::YFP* | 13.1±0.2 | +6% | 82/120 | 0.1174 (<0.0001*^Q35 vhl-1^*) |  |
| *Q35::YFP* control | 13.3±0.3 |  | 58/120 |  |  |
| *elc-1(RNAi); Q35::YFP* | 14.0±0.3 | +6% | 51/120 | 0.1691 |  |
| *vhl-1(RNAi); Q35::YFP* | 14.9±0.2 | +13% | 33/120 | <0.0001 |  |
| *hif-1(ia4); Q35::YFP* control | 15.0±0.3 | +13% | 72/120 | <0.0001 |  |
| *elc-1(RNAi); hif-1(ia4); Q35::YFP* | 14.9±0.2 | +13% | 79/120 | 0.6694 (0.0114 *^Q35 elc-1^*) |  |
| *hif-1(ia4); vhl-1(RNAi); Q35::YFP* | 14.9±0.3 | +12% | 77/120 | 0.8682 (0.5285 *^Q35 vhl-1^*) |  |
| *Aβ* control | 12.0±0.2 |  | 95/120 |  |  |
| *elc-1(RNAi); Aβ* | 13.0±0.2 | +8% | 51/120 | <0.0001 |  |
| *vhl-1(RNAi); Aβ* | 13.1±0.2 | +9% | 74/120 | <0.0001 |  |
| *Aβ* control | 12.0±0.2 |  | 94/120 |  | Fig. 5D |
| *elc-1(RNAi); Aβ* | 12.7±0.2 | +6% | 63/120 | <0.0001 | Fig. 5D |
| *vhl-1(RNAi); Aβ* | 12.4±0.2 | +3% | 73/120 | 0.0001 | Fig. 5D |
| *Aβ* control | 12.9±0.3 |  | 83/120 |  |  |
| *elc-1(RNAi); Aβ* | 13.1±0.2 | +1% | 71/120 | 0.3542 |  |
| *vhl-1(RNAi); Aβ* | 14.2±0.1 | +10% | 54/120 | <0.0001 |  |

In this table, the paralysis assay data sets within solid lines were done in parallel and statistical analysis was done within the sets. Differences in conditions were distinguished by dotted lines. *p* values were calculated within the sets, and were calculated by using the log-rank (Mantel-Cox) method.

Percent changes of mean motility of control RNAi-treated transgenic animals in the *hif-1* mutant background were calculated against those of control RNAi-treated transgenic animals in the wild-type background. Percent changes of mean motility of RNAi-treated transgenic animals in the wild-type and mutant background were calculated against the control immediately above.

*p* values for each condition were calculated against the control immediately above.

Interestingly, we found that *hif-1(ia4)* mutations delayed the paralysis of *Q35::YFP*-expressing transgenic animals in three out of five trials. Since *hif-1(ia4)* mutants have increased activity of DAF-16/FOXO (Leiser *et al.,* 2011), a key longevity transcription factor in *C. elegans*, this moderately delayed paralysis by *hif-1* mutations may be due to the enhanced DAF-16 activity.

**Supplementary References**

Fraser AG, Kamath RS, Zipperlen P, Martinez-Campos M, Sohrmann M, Ahringer J (2000). Functional genomic analysis of *C. elegans* chromosome I by systematic RNA interference. *Nature*. **408**, 325-330.

Kamath RS, Fraser AG, Dong Y, Poulin G, Durbin R, Gotta M, Kanapin A, Le Bot N, Moreno S, Sohrmann M, Welchman DP, Zipperlen P, Ahringer J (2003). Systematic functional analysis of the *Caenorhabditis elegans* genome using RNAi. *Nature*. **421**, 231-237.

Lee SJ, Hwang AB, Kenyon C (2010). Inhibition of respiration extends *C. elegans* life span via reactive oxygen species that increase HIF-1 activity. *Current biology*. **20**, 2131-2136.

Leiser SF, Begun A, Kaeberlein M (2011). HIF-1 modulates longevity and healthspan in a temperature-dependent manner. *Aging cell*. **10**, 318-326.

Mehta R, Steinkraus KA, Sutphin GL, Ramos FJ, Shamieh LS, Huh A, Davis C, Chandler-Brown D, Kaeberlein M (2009). Proteasomal regulation of the hypoxic response modulates aging in *C. elegans*. *Science (New York, N.Y.)*. **324**, 1196-1198.
